# Geometric explanation of the rich-club phenomenon in complex networks

**DOI:** 10.1038/s41598-017-01824-y

**Published:** 2017-05-11

**Authors:** Máté Csigi, Attila Kőrösi, József Bíró, Zalán Heszberger, Yury Malkov, András Gulyás

**Affiliations:** 1 MTA-BME Information Systems Research Group, Department of Telecommunications and Media Informatics, Budapest University of Technology and Economics, H-1117, Budapest, Magyar tudósok krt. 2 Hungary; 2 Federal state budgetary institution of science, Institute of Applied Physics of the Russian Academy of Sciences, 46 Ul’yanov Street, 603950 Nizhny Novgorod, Russia

## Abstract

The rich club organization (the presence of highly connected hub core in a network) influences many structural and functional characteristics of networks including topology, the efficiency of paths and distribution of load. Despite its major role, the literature contains only a very limited set of models capable of generating networks with realistic rich club structure. One possible reason is that the rich club organization is a divisive property among complex networks which exhibit great diversity, in contrast to other metrics (e.g. diameter, clustering or degree distribution) which seem to behave very similarly across many networks. Here we propose a simple yet powerful geometry-based growing model which can generate realistic complex networks with high rich club diversity by controlling a single geometric parameter. The growing model is validated against the Internet, protein-protein interaction, airport and power grid networks.

## Introduction

**Figure 1:**
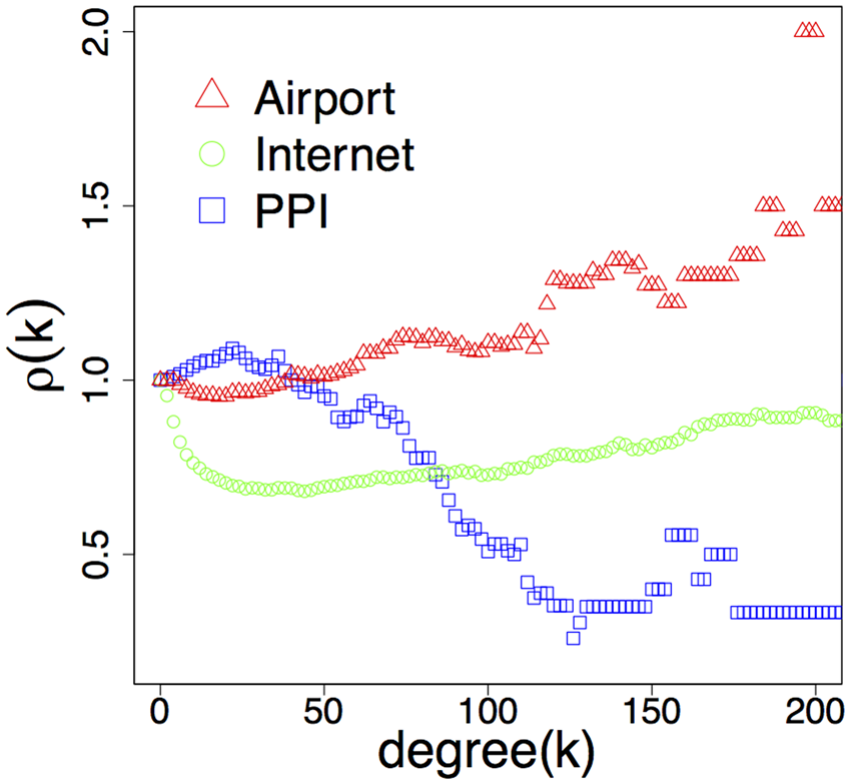
Illustration of the diverse rich club organization in real networks. The plot shows the normalized rich club coefficient *ρ*(*k*) as the function of degree *k* as: $\rho (k)=\frac{\varphi (k)}{{\varphi }_{{\rm{unc}}}(k)},{\rm{where}}\,\varphi (k)$ is the density of the subgraph *G*_*k*_ of the network containing only the nodes with degree > *k*. The *ϕ*_unc_ is the same for the maximally randomized version of the network conserving the degree distribution. One can see that the subgraph of hubs in the airport network possesses about 1.5 times more links than in the randomized version. On the contrary, the hubs in the PPI network have less than the half of the connections experienced in its randomized counterpart.

The rich club organization plays a central role in the structure and function of networks^1–8^. Some networks (e.g. the human brain^7^, airport networks, social networks^1^ and the Internet^8^) have a strong rich club, meaning that their hubs are densely connected to each other. Others (e.g. protein-protein interaction networks^1^, the power grid^9^) behave quite the contrary as the subgraphs made out of their hubs are very sparse. This high variation across networks is illustrated in Fig. 1, which shows the normalized rich club coefficient *ρ*(*k*)^1^ as the function of degree *k* for the airport network, the Internet and the protein-protein interaction network. The explanation and reproduction of this great rich club diversity is highly non trivial. The state-of-the-art models targeting the rich club organization are based on heavy randomization techniques^10–13^, which shuffle network connections until a given organization structure is artificially imitated. Although these randomization-based models are fairly usable, they do not give deeper insight into the mechanisms causing this diversity during the evolution of the networks. Consequently, *growing* models capable of incorporating various rich club networks in a simple and intuitive manner would be useful towards deeper understanding of the underlying evolutionary reasons of this diversity.

Here we propose a simple geometry-based *growing* model which can explain the emergence of the rich club variability in real networks by adjusting a single spatial parameter. Our model is built upon the real-world observation that in some networks the establishment of very long connections is not feasible. For example, in power grid networks, the electric current cannot be transferred efficiently (i.e. without huge losses of energy) over large distances without intermediate transformations at middle stations^14, 15^. Similarly, optical networks apply signal re-generators for the transmission of light signals over large distances to be able to sustain the signal-to-noise ratio^16^. Also in certain social networks, middlemen as intermediate nodes may play crucial role in enhancing cooperation between the individuals or groups^17^. Such networks seem to implement an “artificial” threshold above which no direct connections are allowed. Other networks do not have such inherent thresholds and the length of the edges is only limited by the “natural” geometric boundary of the network. For example, in airport networks we can find very long links, because transferring passengers over large distances is not an issue with the current aviation technologies.

In this paper we confine these observations into a simple geometric growing model, in which we introduce a length threshold for creating edges. We show that such a growing model can naturally reproduce and account for the experienced diversity in the rich-club organization of networks, while keeping other network statistics (diameter, degree distribution and clustering) intact. The applied geometric representation of networks is an active and quickly advancing research direction in network science^18^. There are numerous studies describing networks as random geometric graphs, performing some functions^19, 20^ (e.g. navigation, information transmission) or structural properties (e.g. small-world, clustering, modularity)^21, 22^ of networks in a geometric context, and disclosing some fundamental relations between topology and hidden metric spaces^23^. A the proper choice of geometry (e.g. Euclidean, Bolyai-Lobachevskian hyperbolic geometry or other metric space) can also promote the interpretation of numerous network processes^24–26^.

## Results

**Figure 2:**
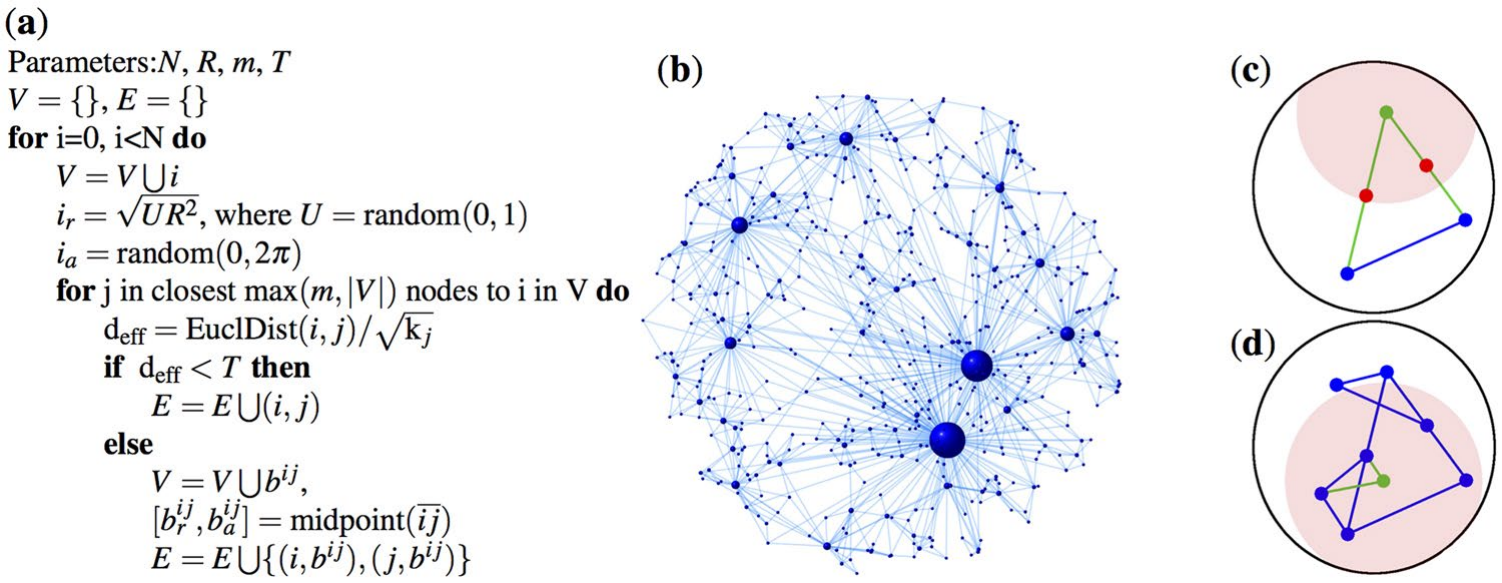
Details of the model. Panel (a) shows the exact pseudocode of the generation process. Panel (b) plots a sample network generated by the model. Panels (c,d) shows the time evolution of the network for *m* = 2. In panel (c) a green node is added to a network consisting the two blue nodes only. The red circle represents the threshold around the new node. As neither of the two blue points reside within the red circle two bridge nodes (red) are inserted to the midpoints of the edges. Panel (d) shows the same network in a later time instant. In this later phase the new green node can connect directly (bridge nodes are not needed) to the two nearest blue nodes as they are closer than *T*.

In our model *N* nodes are randomly generated one after another on an Euclidean 2D *R*-disk with uniformly distributed coordinates. When adding a new node, it selects the *m* closest nodes already residing on the disk (if there are less than *m* nodes on the disk then it selects all of them). The distances between the new node and the old ones are calculated by the Euclidean distances normalized by a function of the old node degrees (as in the Growing Homophilic model^22^). If this “effective” distance between the new node and a selected one is smaller than the threshold *T* then they are directly connected, otherwise a so-called “bridge” node to the midpoint of the two nodes is established and connects to both nodes. The formal description of the network generation process is performed in panel (a) of Fig. 2, while panel (b) shows a small network generated with the model.

Time evolution of the model, as new nodes are inserted into the network at different stages is shown in Fig. 2. For the sake of simplicity, in this illustration the distance normalization by node degrees is omitted. At the beginning of the generation process, many bridge nodes are inserted as the distance between the nodes is typically larger than *T* (see panel (c) in Fig. 2). As the network grows, the average node density and degrees increases, so the typical normalized distance between the nodes will fall below *T* and no more bridge nodes are added (panel (d) in Fig. 2). From this stage the model falls back to the growing homophilic model analyzed in ref. 22. Setting *T* to a very large value (e.g. *T* > 2*R*) completely recovers the model in ref. 22 because bridge nodes are never inserted to the graph. We show, that by varying *T*, the model generates complex networks with diverse rich-club organization, while having scale-free degree distribution, small diameter and large clustering. In the remaining of the paper we will use the settings summarized in Table 1 in our analytical and simulation results.

**Table 1:** Simulation settings.

Network	*N*	*m*	*R*	*T*
Generated *T* = 12	5000	3	50	12
Generated *T* = 30	5000	3	50	30
Generated *T* = 100	5000	3	50	100

### Number of bridge nodes

First, we show that the total number of bridge nodes quickly converges to a relatively small value compared to the network size (*N*) during the generation of the graph, and this value is independent of the graph size. To support this observation we give a recursive estimation of the expected number of new bridge nodes generated at each step of the model, and based on this recursion a mathematical expression is given to the limit of the expected total number of bridge nodes (see Methods for more details). By analyzing the recursion one can show that the expected number of bridge nodes at step *N* denoted by *b*_*N*_ can approximately be expressed in the form

1 $${b}_{N}\approx \exp (-{f}_{1}N+{f}_{2}{\rm{l}}{\rm{o}}{\rm{g}}N+{f}_{3}),$$

where the functions *f*_1_, *f*_2_ and *f*_3_ may depend on *R*, *T*, *m* but are independent from *N*. From this it immediately follows, that for the total number of bridge nodes *B*_*N*_

2 $${B}_{N}\approx {\int }_{x\mathrm{=1}}^{N}\exp (\,-\,{f}_{1}x+{f}_{2}\,\mathrm{log}\,x+{f}_{3}){\rm{d}}x\to \exp ({f}_{3}){E}_{-{f}_{2}}({f}_{1}){\rm{as}}\,N\to \infty $$

where *E* is the exponential integral function. The vanishing term during the convergence in *B*_*N*_ is ${N}^{1+{f}_{2}}{E}_{-{f}_{2}}({f}_{1}N)$ and also approximately exponential.

**Figure 3:**
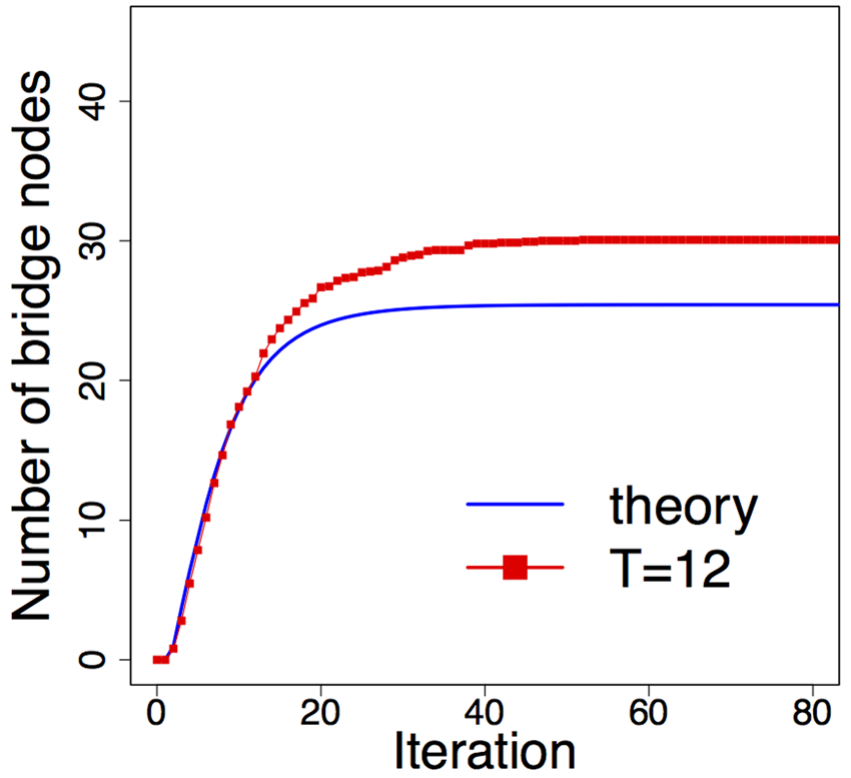
The recursively estimated number of bridge nodes in each iteration is plotted with the simulated values. The *T* = 12 line shows the average of 15 simulation runs.

In Fig. 3 the expected total number of bridge nodes (*B*_*N*_) calculated by recursion (9) is plotted in each iteration together with the simulation result for the same parameters. The two plots readily justify that *B*_*N*_ has a characteristic plateau after certain iterations, which means that *B*_*N*_ converges to a finite fixed value during the graph generation process. This also illustrates that for sufficiently large networks the total number of bridge nodes is negligible comparing to the network size. Furthermore, according to statistical tests the overall distribution of the nodes on the *R*-disk is apparently not affected by the existence of the bridge nodes, and still can be treated as uniform.

### Diameter, clustering and degree distribution

**Figure 4:**
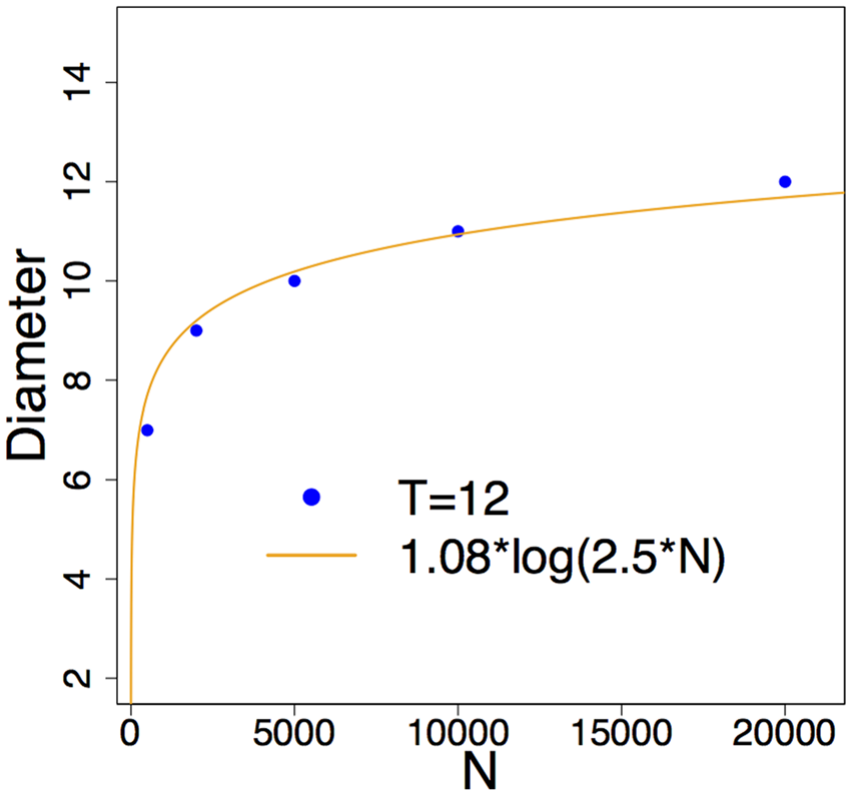
Small world property of *T* = 12 networks. The diameter of the network grows logarithmically with the number of nodes.

The diameter of all three generated networks (see Table 1) is around 9–10, similar to the real networks (Table 2). Figure 4 shows that the diameter of the *T* = 12 networks is an approximate logarithmic function of the network size, which confirms the small-world property. Also the generated networks have high clustering coefficients with values very close to that of real networks. Finally, Table 2 confirms that the clustering coefficient is insensitive to the threshold parameter. Now we show that the generated networks has scale-free degree distribution independently of *T*.

**Table 2:** Basic topological properties of real and generated networks.

Network	N	Edges	Diameter	Avg. dist.	Clustering coefficient
Generated *T* = 12	5030	15024	10	4.54	0.66
Generated *T* = 30	5004	14998	9	4.51	0.67
Generated *T* = 100	5000	14994	10	4.46	0.69
PPI network	5084	22148	10	3.98	0.12
Airport network	2845	10409	10	3.75	0.59
Internet	23748	58414	10	3.52	0.61

**Theorem 1** *The networks produced by the model have scale-free degree distribution with γ* = 3 *when N* → ∞.

**Proof:** Suppose we compute the effective distance as ${d}_{eff}=\frac{{d}_{Euc}}{\sqrt{k}}$. At each insertion step the algorithm connects the new element to exactly *m* neighbors that globally minimize the normalized distance. To infer the degree distribution of the neighbor elements, we temporary fix the distance to the *m* + 1-th nearest neighbor ${d}_{{\rm{eff}}}^{m+1}$ and randomly shuffle positions of the *m* neighbor nodes under the condition that they all remain the *m* nearest neighbors with respect to the new element (i.e. having effective distance to the new element less than ${d}_{{\rm{eff}}}^{m+1}$).

For every possible value of the neighbor degree *k*, possible element positions are bounded in the initial Euclidean space by a radius *r*_Euc_ = ${d}_{{\rm{eff}}}^{m+1}\sqrt{k}$. Since the nodes are distributed uniformly in the Euclidean space, the probability of having an element with degree k proportional to the *r*_Euc_-ball volume. Thus, under fixed ${d}_{{\rm{eff}}}^{m+1}$ the overall probability of connecting to an element with degree *k* is proportional to (*k*).

The probability inferred for a fixed value of ${d}_{{\rm{eff}}}^{m+1}$ does not depend on either the value of ${d}_{{\rm{eff}}}^{m+1}$, or the positions of the nodes that are not the closest neighbors of the inserted elements, so that is true for every possible positions of the elements in the Euclidean space and overall probability of connection to a node is proportional to its degree (*k*). This means that new nodes connect to the old ones with probability proportional to *k*, which is equivalent to the Barabasi-Albert model^27^, proved to produce scale-free networks with *γ* = 3.

**Figure 5:**
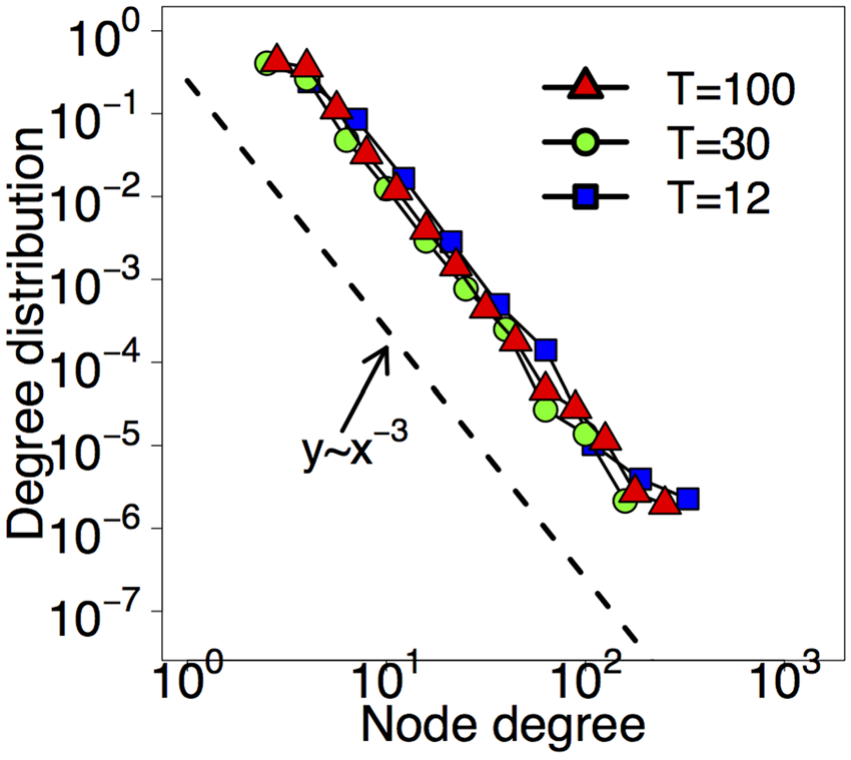
The degree distributions of our simulated networks. The plot confirms that the choice of *T* does not effect the degree distribution, which is a power-law with *γ* = 3.

The Fig. 5 shows the degree distributions of three networks generated with our model with various values of *T*. The plot readily confirms that the degree distributions are indeed scale-free with *γ* = 3 independently of *T*.

### Rich-club coefficient

**Figure 6:**
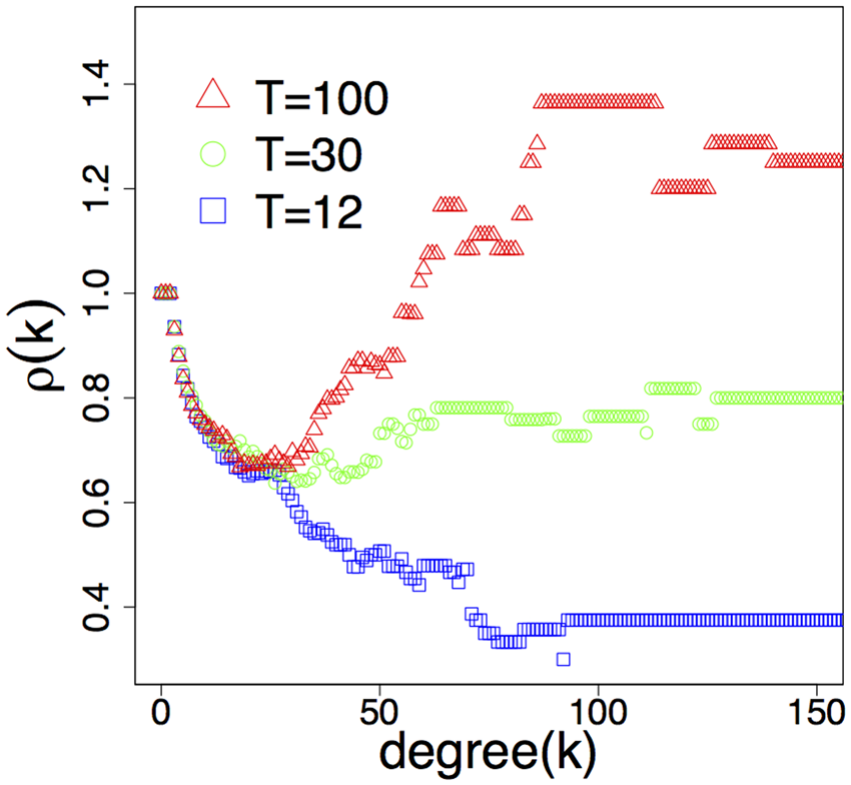
Rich-club organization in networks generated by the model with different settings of *T*. The plot readily confirms that our model is able to generate rich-club diversity by simply adjusting *T*. We note the remarkable similarity with the same plot for real networks in Fig. 1.

Although the insertion of bridge nodes keeps degree distribution, clustering and diameter intact, the simulation results plotted in Fig. 6 clearly show that the graphs generated by the model differ greatly in their rich-club organization depending on *T*. Setting *T* to the diameter of the *R*-disk (*T* = 100, red triangles in Fig. 6), the model does not limit the lengths of the edges artificially, so the only limiting factor is the natural geometry of the disk itself. In this case we obtain a network with a strong rich-club, similarly to the airport network. Conversely, adjusting *T* to 12, the model will create only edges having *d*_eff_ < 12. This is a strong “artificial” limitation for the edge lengths imposed by the generation process. As a result, the model yields a network with no rich-club (*T* = 12, blue squares in Fig. 6), likewise the PPI network. We note the appealing similarity between Figs 1 and 6, showing the rich-club diversity in real networks.

## Discussion

**Figure 7:**
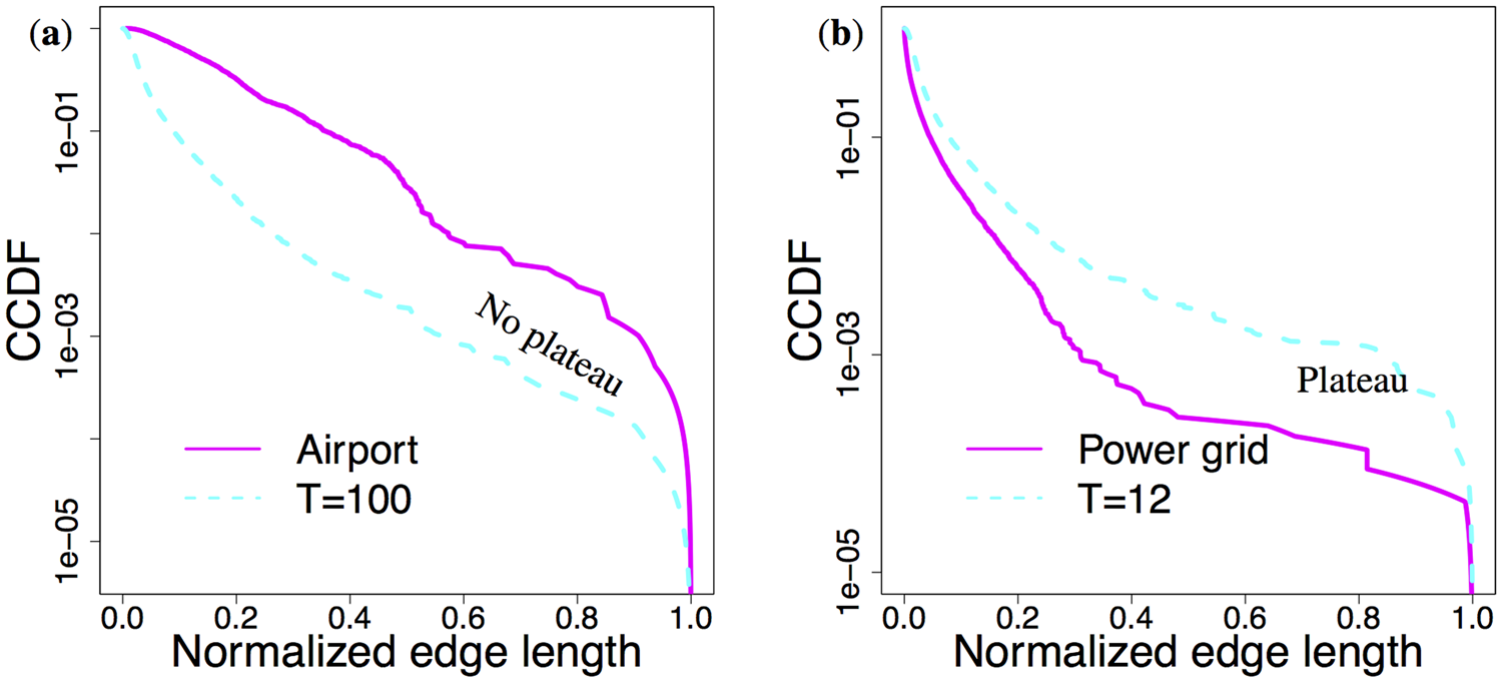
Edge length distribution in networks with different rich club organization. While panel (a) shows a continuously decreasing length distribution in case of rich-club networks, there is a clearly visible plateau before the cutoff of the CCDF function in networks having no rich-clubs (panel (b)).

An intriguing question could be whether our model captures something fundamental from the growth processes of real networks, or exhibit similar rich-club diversity simply by chance. To answer this question we have performed the CCDF’s (Complementary Cumulative Distribution Function) of the normalized edge length distribution in a rich-club (airports with flights in the US) and a non rich-club network (the North American Power Grid) together with the networks generated with our model in Fig. 7. Panel (a) shows continuously significant (on all length scale) decrease of edge length distributions before the final “natural” cutoff for the airport and the *T* = 100 networks caused by the geometry of the continent and the *R*-disk respectively. On panel (b) however we can observe a clearly visible plateau before the cutoff of the edge lengths in the power grid network. This means that edge lengths are much denser near the cutoff, which in this case is rather “artificial” and caused by the growth process of the network and not the underlying geometry. Our model produces a very similar edge length distribution for the setting *T* = 12. These results hint that networks with no rich-clubs have a very similar limiting for the length of the connections as our model does. As a consequence, this length-limiting phenomenon can also account for the emergence of the observed diverse rich-club organization in real networks.

These two examples also underline that our method is parsimonious in a sense that the rich club organization can be tuned by only a single geometric threshold parameter in a growing homophilic model. We think the results presented in this paper are strong indications that the rich club diversity can be placed at all on a growing/evolutionary perspective, and provide deeper insight into the mechanisms resulting certain rich club behavior during the growth of networks.

## Methods

### Recursive estimation of the number of bridge nodes

Let *A*(*r*, *T*, *R*) be the area of the intersection of an *r*–centered disk with radius *T* and the *R*–disk, and let *p*(*r*, *T*, *R*) be the fraction of *A*(*r*, *T*, *R*) and the area of the *R*–disk, i.e. $p(r,T,R)=\frac{A(r,T,R)}{{R}^{2}\pi }$. Further, let us assume that there are already *j* nodes in the network. The (*j* + 1)^th^ randomly generated node will connect to the *m* nearest neighbors. For calculating the necessary bridge nodes in this step, the task is to determine what are the nodes among the *m* nearest neighbors which are farther than *T*. To ease the computation, the degrees of the neighbors are substituted by their expectation values (denoted by ${\overline{k}}_{j}$ and to be determined later). Since the effective distance is computed as the Euclidean distance divided by $\sqrt{{\overline{k}}_{j}}$, it is approximately equivalent to investigate the expected number of points among the *m* nearest ones being outside of the (*j* + 1)^th^ node $T\sqrt{{\overline{k}}_{j}}$- radius vicinity. This will be equal to the expected number of newly inserted bridge nodes at this step. Denote the radial coordinate of the (*j* + 1)^th^ node by *r* and assume that the previously generated random points and established bridge nodes are still evenly distributed on the *R*–disk. With this, the probability that *i*, 0 ≤ *i* ≤ *j* nodes among the *j* ones are closer to the (*j* + 1)^th^ node than $T\sqrt{{\overline{k}}_{j}}$ is

3 $$(\begin{array}{c}j\\ i\end{array})p{(r,T\sqrt{{\bar{k}}_{j}},R)}^{i}{(1-p(r,T\sqrt{{\bar{k}}_{j}},R))}^{j-i}$$

and hence the expected number of necessary bridge nodes at this step is

4 $$\sum _{i\mathrm{=0}}^{\min (m-\mathrm{1,}j-1)}\,{\rm{\min }}(m-i,j-i)(\begin{array}{c}j\\ i\end{array})p{(r,T\sqrt{{\bar{k}}_{j}},R)}^{i}{(1-p(r,T\sqrt{{\bar{k}}_{j}},R))}^{j-i}:={\beta }_{j}(r,T\sqrt{{\bar{k}}_{j}},R,m)$$

Note, that this is still a conditional expectation value which is to be de-conditioned by the density of the radial coordinate *r*. Towards the de-conditioning, first the function *A*(*r*, *T*, *R*) is to be determined. Clearly, *A*(*r*, *T*, *R*) = *T* ^2^ *π* if *r* ≤ *R* − *T*, i.e. there is no intersection of the two disks. Otherwise, if *r* ≥ *R* − *T* then by using straightforward geometrical calculations

5 $$A(r,T,R)=\alpha {R}^{2}+\gamma {T}^{2}-2\sqrt{s(s-R)(s-T)(s-r)}$$

where

6 $$\alpha =\arccos \frac{{R}^{2}+{r}^{2}-{T}^{2}}{2rR},\gamma =\arccos \frac{{r}^{2}+{T}^{2}-{R}^{2}}{2rT},s=\frac{R+T+r}{2}\mathrm{.}$$

Now, the de-conditioning is possible with *p*(*r*, *T*, *R*) = *A*(*r*, *T*, *R*)/(*R* ^2^ *π*) and the density of the radial coordinate *r*, which is $\frac{2r}{{R}^{2}}$. Further, let *j*(*N*) = *N* + *b*_1_ + *b*_2_ + … +*b*_*N*_ where *N* is the randomly generated points and *b*_*l*_ *l* = 1, …, *N* is the expected number of bridge nodes established after the *l* ^th^ random node. For completing the recursive estimation, the expected degree should also be expressed upon the *l* ^th^ random node generation. This is

7 $${\bar{k}}_{l}=\frac{2(lm-\frac{m(m+\mathrm{1)}}{2}+2\sum _{i\mathrm{=1}}^{l}{b}_{i})}{l+\sum _{i\mathrm{=1}}^{l}{b}_{i}},{\rm{for}}\,l > m,$$

else

8 $${\bar{k}}_{l}=\frac{2(\frac{l(l-\mathrm{1)}}{2}+2\sum _{i\mathrm{=1}}^{l}{b}_{i})}{l+\sum _{i\mathrm{=1}}^{l}{b}_{i}}\mathrm{.}$$

For *l* = 1 let *b*_1_ = 0, ${\overline{k}}_{1}=0$ and let ${B}_{N}={\sum }_{i=1}^{N}{b}_{i}$. The main recursion can now be expressed as

9 $$\,\,\,\,\,\,\,\,\,\,\,\,{b}_{N+1}={\int }_{r\mathrm{=0}}^{R}{\beta }_{j(N)}(r,T\sqrt{{\bar{k}}_{j(N)}},R,m)\frac{2r}{{R}^{2}}{\rm{d}}r\mathrm{.}$$

### Data Availability

The data that support the findings of this study are available from public data repositories. In particular, the topology of the AS level Internet has been downloaded from CAIDA (Center for Applied Internet Data Analysis, www.caida.org). We have downloaded the airport network from the OpenFlights database (www.openflights.org). We used the DIP^28^ database as a source for the protein-protein interaction network of the S. cerevisiae. Finally, the map of the north american power grid has been downloaded from ref. 29.
